# Supplementary material for: Evolution of mechanisms controlling epithelial morphogenesis across animals: new insights from dissociation-reaggregation experiments in the sponge Oscarella lobularis
Source: BMC Ecol Evol. 2021 Aug 21;21:160. doi: 10.1186/s12862-021-01866-x (PMC8380372; doi:10.1186/s12862-021-01866-x)

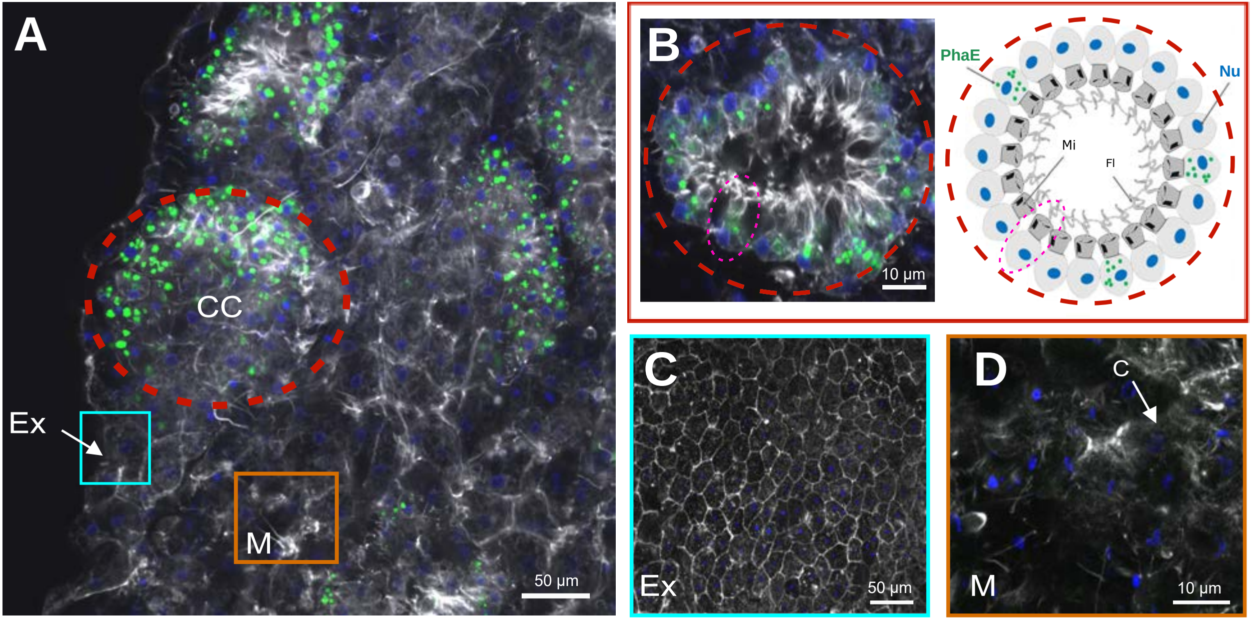
A
CC
Ex
M
50 µm
B
10 µm
PhaE
Nu
Mi
Fl
C
Ex
50 µm
D
C
M
10 µm

Supplementary Figure S2

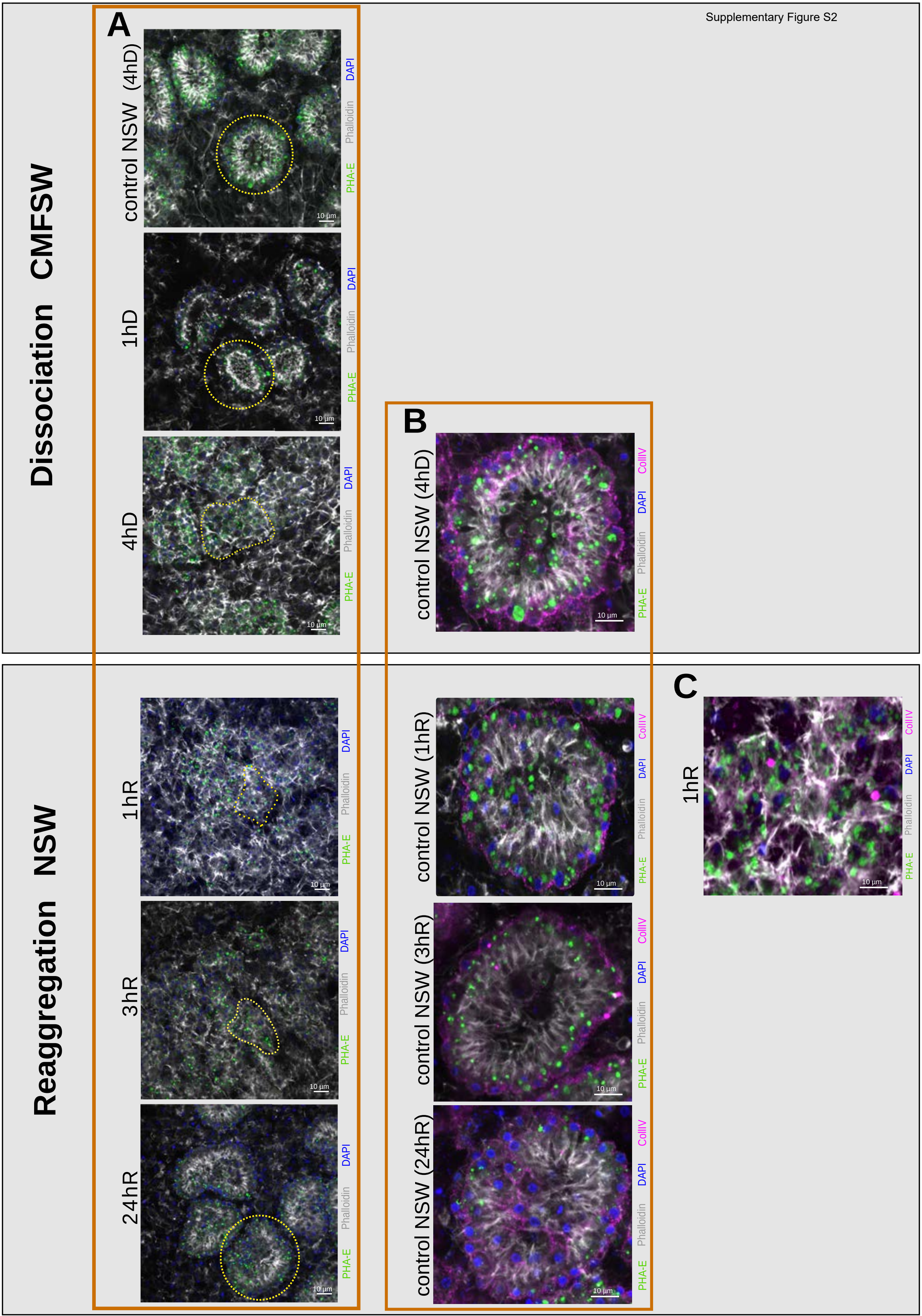

Supplementary Figure S3

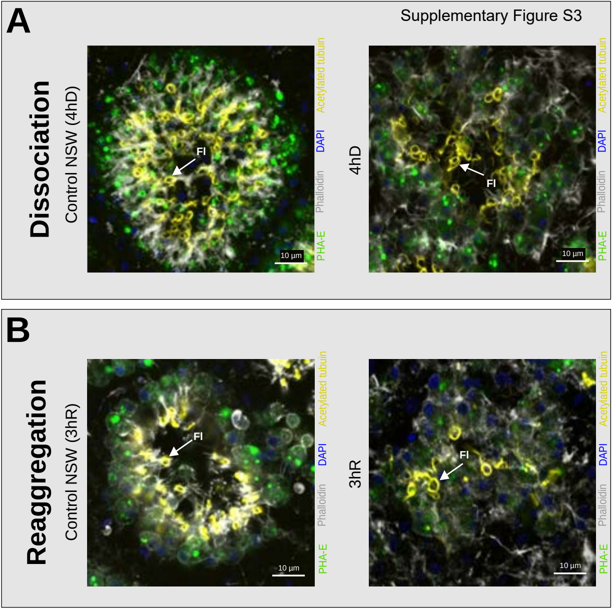

Supplementary Figure S4

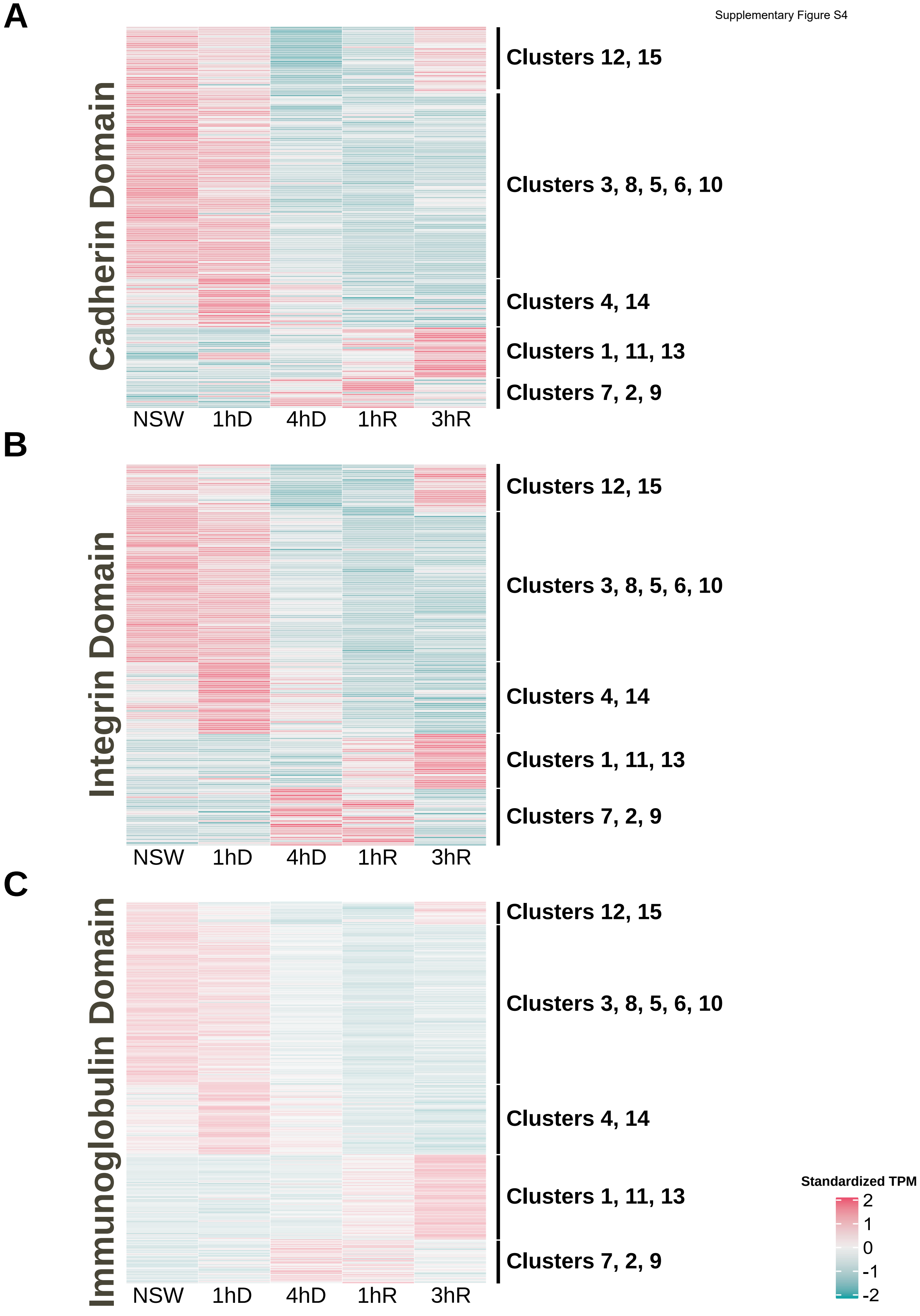

Supplementary Figure S5

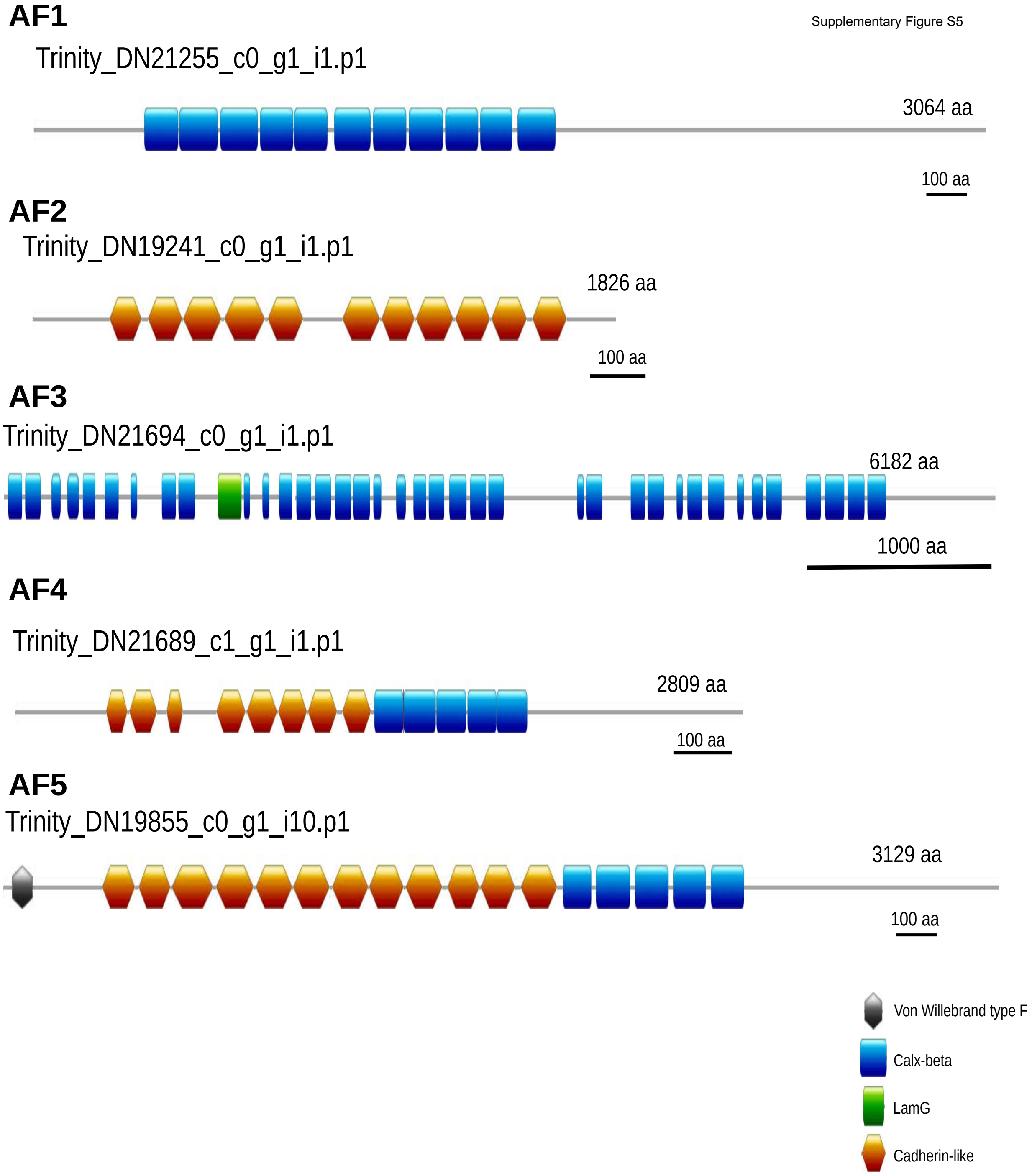

Supplementary Figure S6

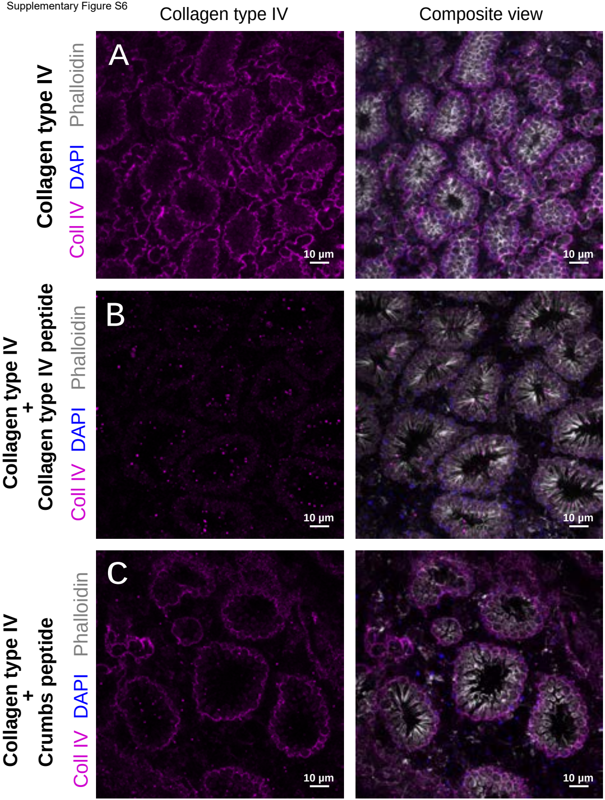

Supplementary Figure S7

A

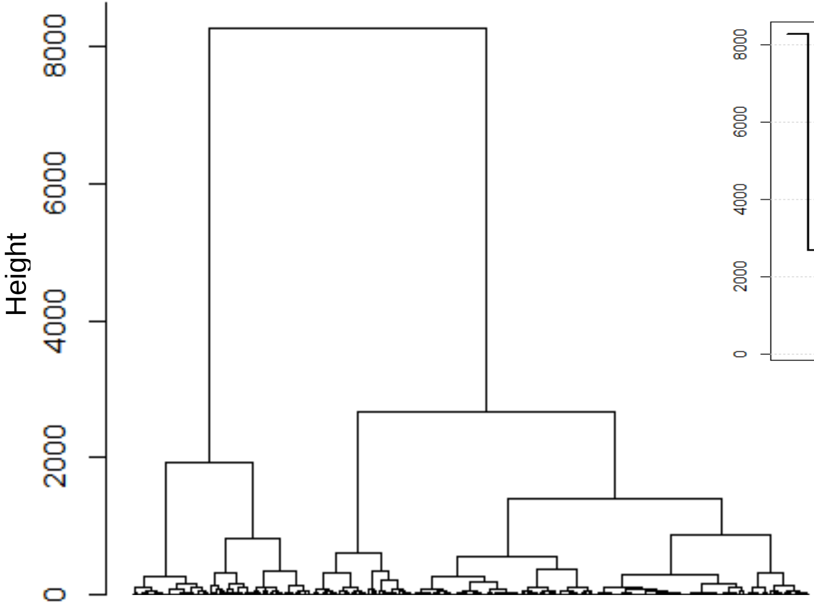

B

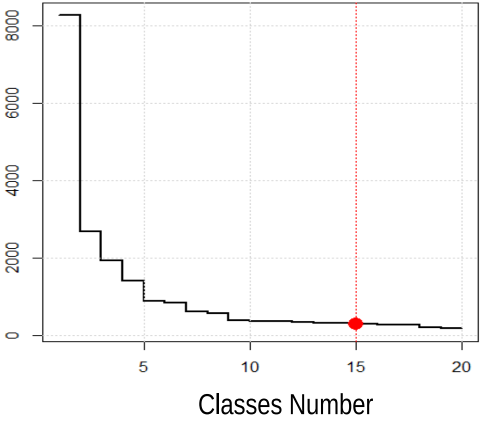

Supplement: Supplementary file 1 — Additional file 1: Figure S1. Specificity of the PhaE staining. [file 12862_2021_1866_MOESM1_ESM.pdf]
