# Supplementary figures and images for: Evolution of mechanisms controlling epithelial morphogenesis across animals: new insights from dissociation-reaggregation experiments in the sponge Oscarella lobularis
Source: BMC Ecol Evol. 2021 Aug 21;21:160. doi: 10.1186/s12862-021-01866-x (PMC8380372; doi:10.1186/s12862-021-01866-x)

# Dissociation CMFSW

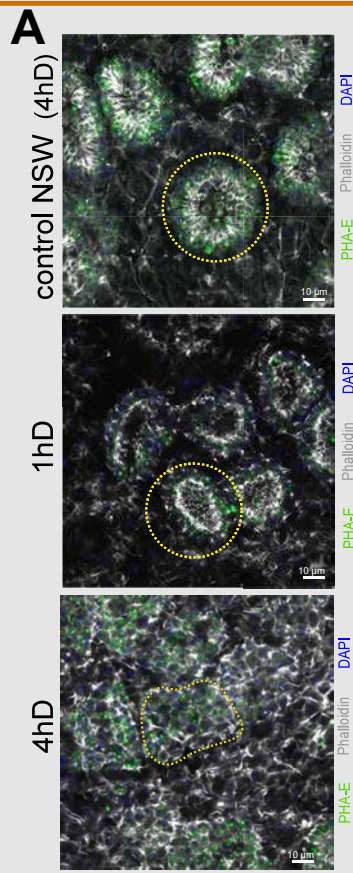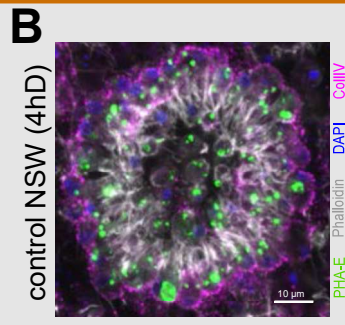

# Reaggregation NSW

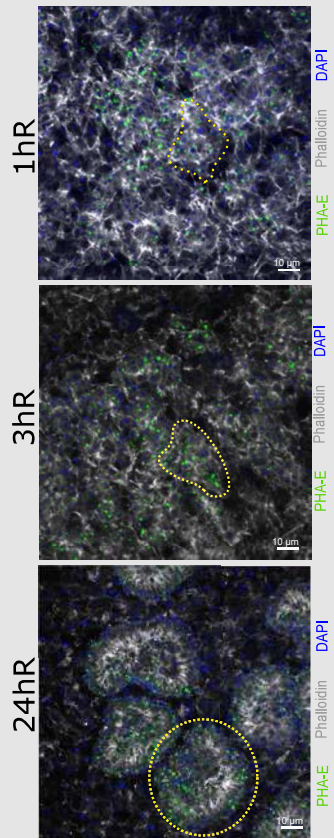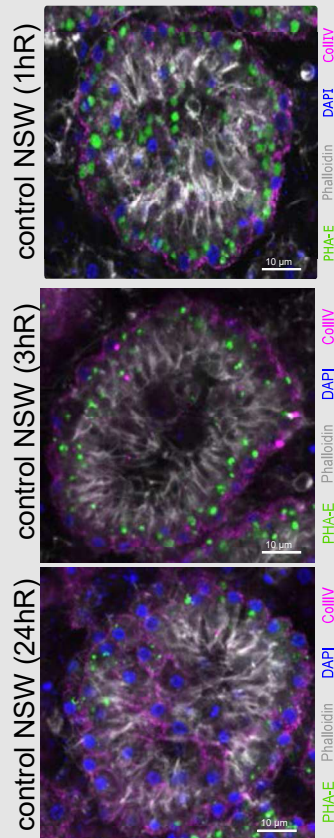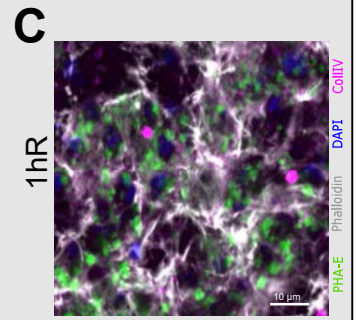

Supplement: Supplementary file 2 — Additional file 2: Figure S2. S Comparison of the aspect of choanocyte chambers under different conditions (confocal microscopy views). [file 12862_2021_1866_MOESM2_ESM.pdf]

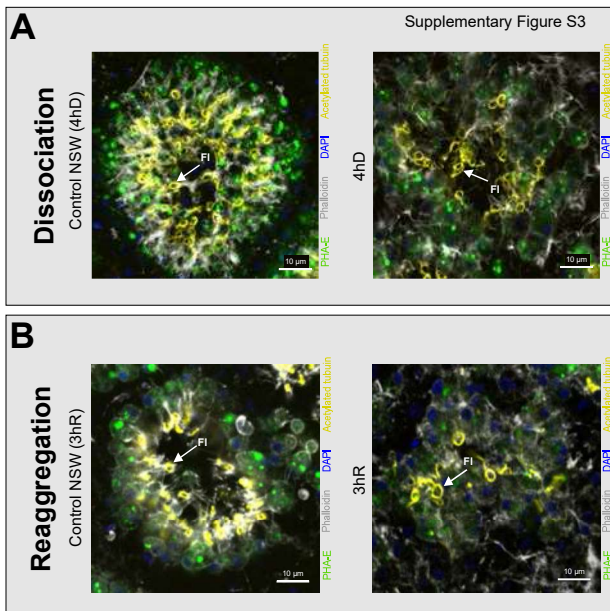

Supplement: Supplementary file 4 — Additional file 4: Figure S3. Acetylated tubulin immunostaining of the choanocyte flagella during dissociation and reaggregation. [file 12862_2021_1866_MOESM4_ESM.pdf]

**A**

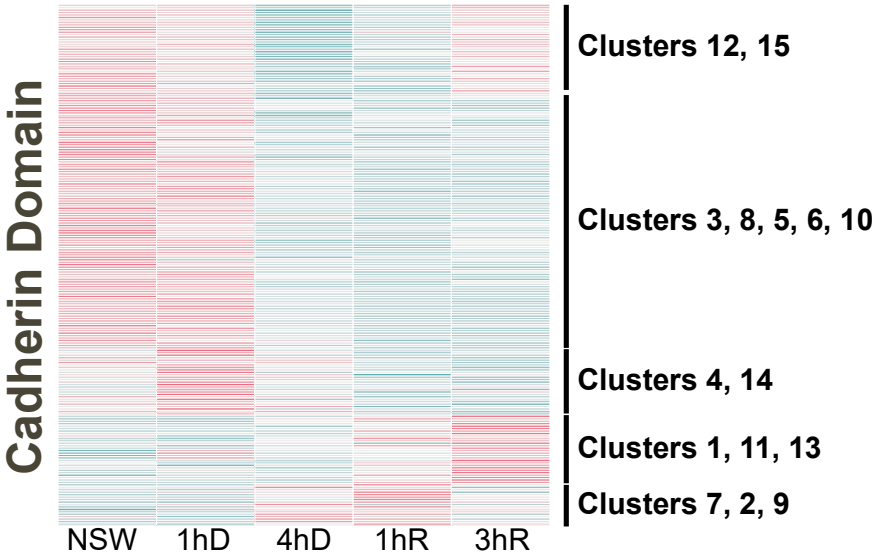

**B**

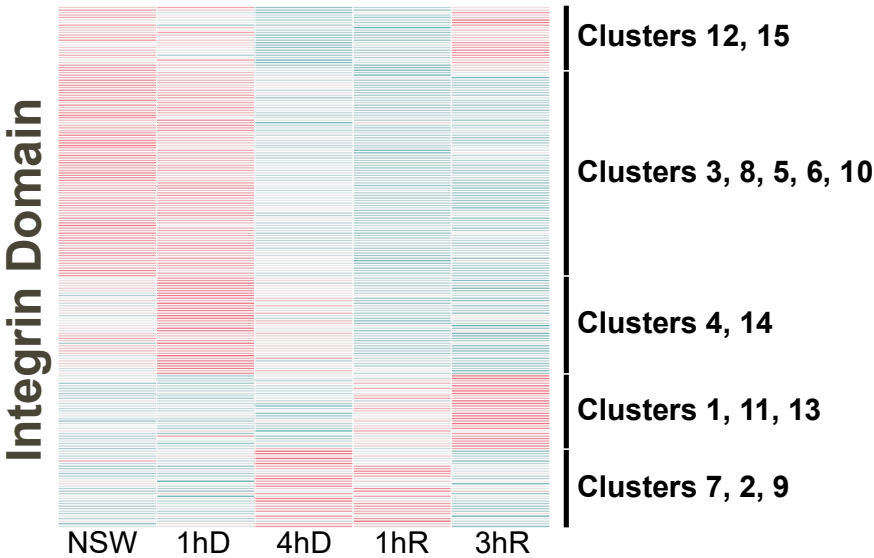

**C**

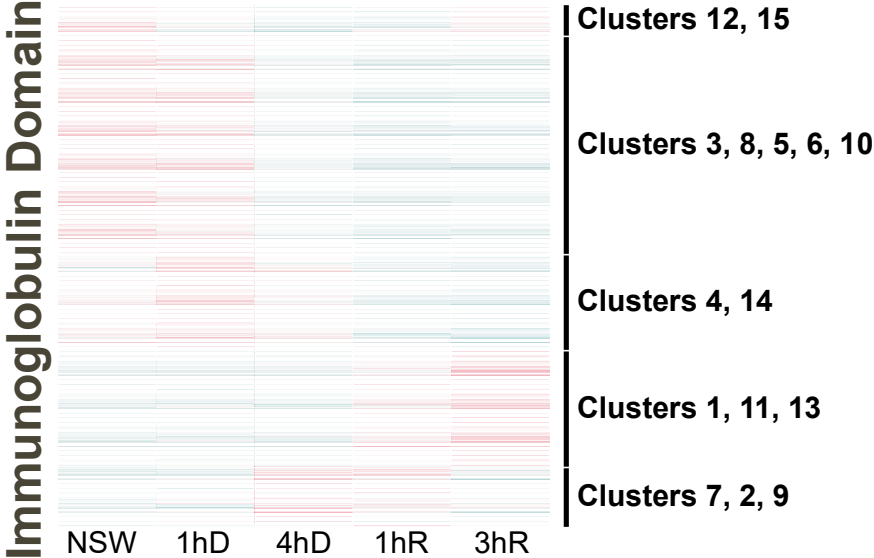

Supplement: Supplementary file 14 — Additional file 14: Figure S4. Distribution of the transcripts with CAMs conserved domain during dissociation and reaggregation. [file 12862_2021_1866_MOESM14_ESM.pdf]

## AF1

Trinity\_DN21255\_c0\_g1\_i1.p1

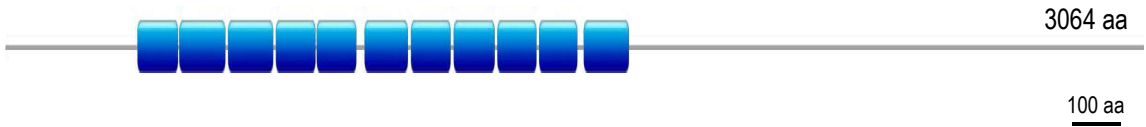

## AF2

Trinity\_DN19241\_c0\_g1\_i1.p1

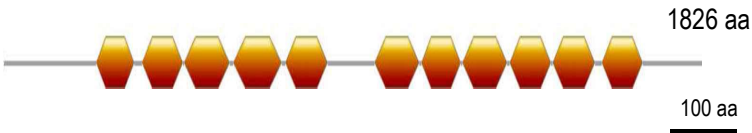

## AF3

Trinity\_DN21694\_c0\_g1\_i1.p1

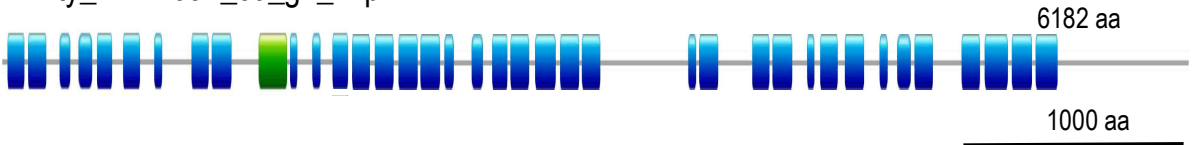

## AF4

Trinity\_DN21689\_c1\_g1\_i1.p1

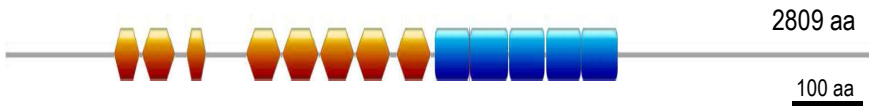

## AF5

Trinity\_DN19855\_c0\_g1\_i10.p1

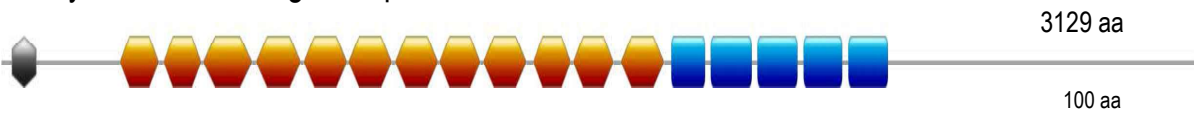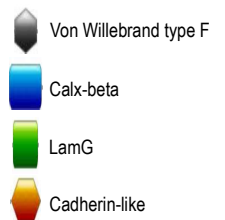

Supplement: Supplementary file 16 — Additional file 16: Figure S5. Domain composition of putative Aggregation Factors in Oscarella lobularis. [file 12862_2021_1866_MOESM16_ESM.pdf]

Collagen type IV

Composite view

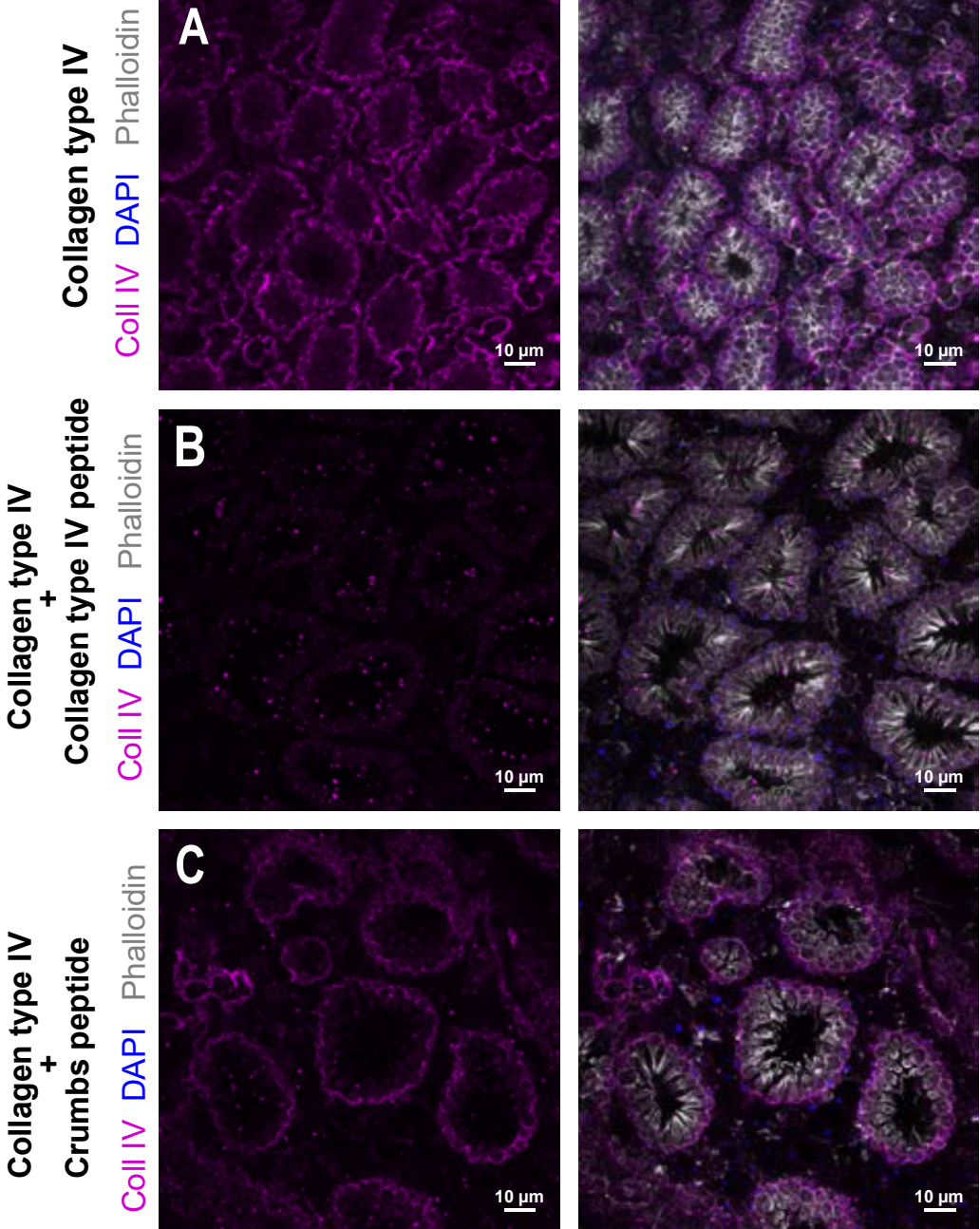

Supplement: Supplementary file 18 — Additional file 18: Figure S6.Validation of the antibody against O. lobularis type IV Collagen by peptide competition assay. [file 12862_2021_1866_MOESM18_ESM.pdf]

**A**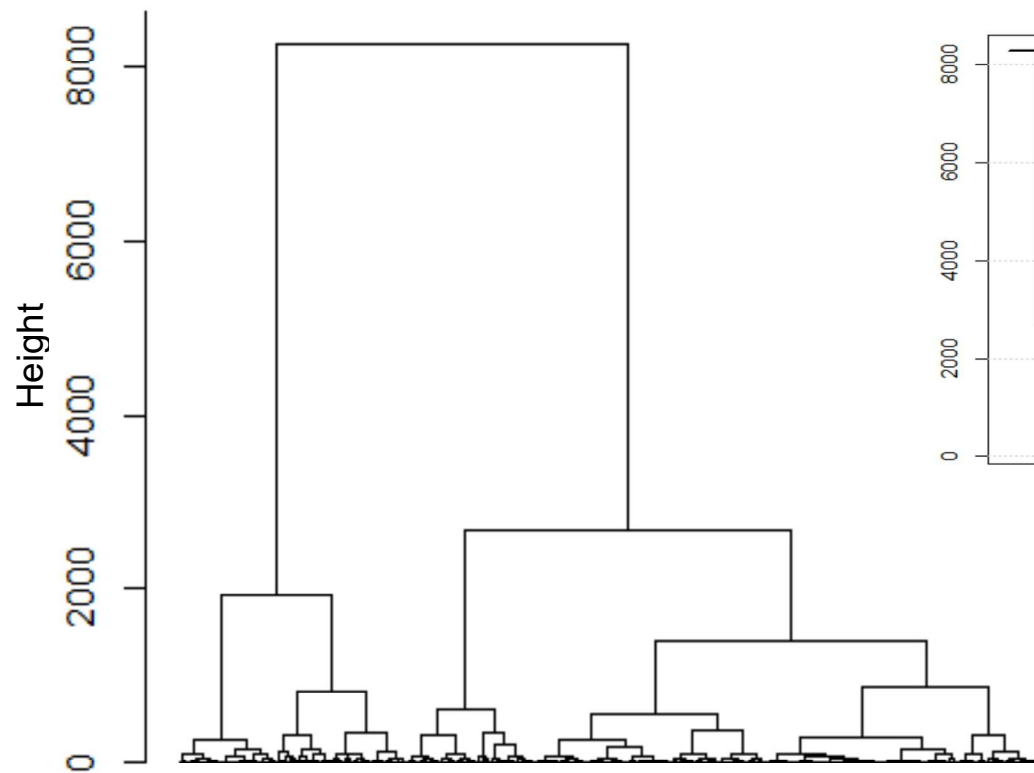**B**

Supplementary Figure S7

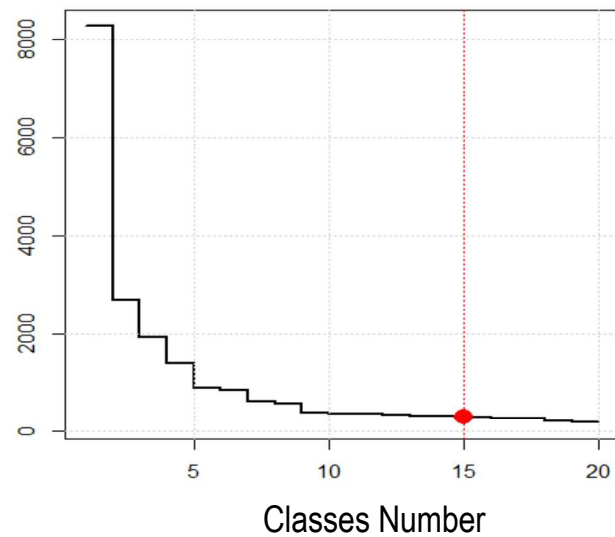

Supplement: Supplementary file 20 — Additional file 20: Figure S7. Hierarchical clustering on TPM values. [file 12862_2021_1866_MOESM20_ESM.pdf]
